# Chemotherapy of Human African Trypanosomiasis

**DOI:** 10.1155/2009/195040

**Published:** 2009-08-20

**Authors:** Cyrus J. Bacchi

**Affiliations:** Department of Biological and Health Sciences, Haskins Laboratory, Pace University, New York, NY 10038, USA

## Abstract

Human Africa trypanosomiasis is a centuries-old disease which has disrupted sub-Saharan Africa in both physical suffering and economic loss. This article presents an update of classic chemotherapeutic agents, in use for >50 years and the recent development of promising non-toxic combination chemotherapy suitable for use in rural clinics.

## 1. Introduction

Human African Trypanosomiasis (HAT) or African trypanosomiasis has been endemic in sub-Saharan Africa for thousands of years. Currently approximately 50 million people are at risk for this disease in an area of 10 million square kilometers [1] HAT is caused by two hemoflagellates of the *Trypanosoma brucei* subgroup, *T. b. gambiense* (West African or Gambian form) and *T. b. rhodesiense* (East African or Rhodesian form). The parasites are transmitted by the tsetse fly between humans and reservoir domestic and wild animals. In the early-stage of the disease, the parasite is found in the bloodstream and lymphatic system. In the later, central nervous system stage, cerebrospinal fluid, and neural tissue become infected. Symptoms of early-stage include fever, chills, headache, and lymphadopathy. In the later CNS stage, severe headaches, insomnia, progressive mental deterioration, psychiatric manifestations, and tremors are common, finally culminating in coma and death [2]. The surface of the trypanosome is covered by variant surface glycoprotein (VSG) which is the main antigenic determinant to the human immune system [3]. The genome contains ~1 000 genes capable of coding for VSG genes which are randomly switched on and off at each generation [4]. This immune evasion mechanism makes it unlikely that a vaccine could be developed for HAT. 

 The attention paid to developing chemotherapy for HAT has lagged behind that for other tropical diseases [5]. The agents in routine clinical use have been available for >50 years and are not ideal drugs, some curing only early-stage disease (pentamidine, berenil or suramin), with the only routinely available drug for late-stage, CNS disease (Melarsprol, Mel B, Arsobal) having significant toxicity [6]. With the exception of suramin, resistance to these agents is growing. This is due in part to development of reduced uptake with melarsoprol and pentamidine [7]. 

Difluoromethylornithine (eflornithine, DFMO, OrnidylR) is the only new useful addition to this list for treatment of late-stage since the early 1950s [7, 8]. A recent development has been the success in clinical trial of a DFMO-nifurtimox combination for CNS disease. This reduces the DFMO dosage and has no significant toxicity [9]. Parafuramidine maleate (DB289) is being developed as an orally administered alternative to intramuscular dosing with pentamidine in hope that it might facilitate treatment of early-stage, non-CNS disease [10]. 

## 2. Current Chemotherapy

Pentamidine is a water-soluble aromatic diamidine that has been in use since the 1930s. It is effective against early-stage *T. b. gambiense* infection, but is less effective against *T. b. rhodesiense* infection, and is ineffective against late-stage disease [10, 11]. Dosing for early-stage disease consists of a series of 7–10 intramuscular injections; however, shorter dose regimens are being explored [10]. African trypanosomes have a nucleoside (adenine/adenosine: P2) transporter that takes up pentamidine, resulting in the concentration of the agent at levels many times that in plasma [12, 13]. Recent studies have found two other transporters, besides P2, that also transport pentamidine and may be responsible for 50% of its uptake [14]. Many studies have focused on the mechanism of pentamidine action; however, none appears to conclusively define the target. It is known to bind to the minor groove of kinetoplast (mitochondrial) DNA, and to promote the cleavage of kinetoplast minicircle DNA, eventually leading to the development of dyskinetoplastic cells. Despite its effects on kinetoplast DNA, pentamidine has no effect on nuclear DNA, and dyskinetoplastic forms can persist in the bloodstream of mammals [10, 11]. Pentamidine was also found to be a reversible inhibitor of S-adenosylmethionine (AdoMet) decarboxylase, an enzyme in the polyamine biosynthetic pathway, but this is unlikely as the primary mechanism of action [10]. Although Ki values were in the 200-*μ*M range, we now know that this internal concentration is achievable via uptake through the P2 nucleoside–pentamidine transporter, and two other recently discovered transporters HAPT1 and LAPT1 [14, 15]. Other targets studied previously in trypanosomes include the inhibition of glycolysis and lipid metabolism, as well as effects on amino acid transport and ion exchange. The fact that pentamidine does not kill trypanosomes outright and bloodstream forms persist after treatment argue for a sustained effect more consistent with interference of parasite nucleic acid metabolism [10, 11]. 

Diminazene aceturate (Berenil) is an aromatic diamidine developed by Hoechst as treatment for bovine trypanosomiasis; however, its apparent low incidence of adverse reactions and significant therapeutic activity has led some physicians in endemic countries to use it extensively. It is effective against early-stage *T. b. gambiense* and *T. b. rhodesiense*. Berenil has also been used in combination with melarsoprol for the late-stage disease. Mechanistically, like pentamidine, berenil has also been linked to kinetoplast DNA binding at the minor groove and cleavage of minicircle DNA. As with pentamidine, berenil may also interfere with RNA editing and *trans*-splicing [11]. Berenil is also a more effective and noncompetitive inhibitor of AdoMet decarboxylase in trypanosomes, resulting in the reduction of spermidine content and elevating putrescine in the parasite. Berenil uptake, as with pentamidine, occurs via the P2 nucleoside transporter, which allows significant accumulation from the external environment. The other pentamidine transporters, HAPT1 and LAPT1 appear to play only a minor role in berenil uptake [14]. Although berenil has been used for many years on thousands of sleeping sickness patients, there is little published on its toxicity [10, 15]. This may in part be due to physicians who are unwilling to document human studies with an agent licensed for veterinary use. However, personal accounts of those using berenil in humans indicate it is well tolerated. 

Suramin is a sulfonated naphthylamine, which has been used successfully against early-stage sleeping sickness caused chiefly by *T. b. rhodesiense*. Suramin does not penetrate the blood-brain barrier and is not used for CNS-stage disease. It was first used in 1922, developed from the closely related azo dyes, trypan red, and trypan blue [6, 10]. Suramin has an extremely long half-life in humans, 44–54 days, the result of avid binding to serum proteins. Suramin binds to many plasma proteins including LDL, which trypanosomes avidly bind and endocytose as a result of specific membrane receptors. LDL is a prime source of sterols for bloodstream trypanosomes [10, 16]. Suramin has been shown to inhibit all of the glycolytic enzymes in *T. b. brucei* and also other enzymes, including those of the pentose phosphate pathway [10]. This specificity for trypanosomal enzymes was attributed to higher (basic) isoelectric points for the parasite enzyme than the mammalian enzymes, allowing the negatively charged suramin to bind preferentially to the parasite enzymes. In practice, because most trypanosome glycolytic enzymes are contained in a membrane-bound cytosolic organelle, the glycosome, it is not likely that rapid massive binding occurs. This would rapidly induce lysis in bloodstream forms that depend on glycolysis as the sole energy-generating source. Rather, animals that are heavily infected with trypanosomes and given suramin show a slow decrease in parasite numbers, indicating that enzyme inhibition occurs slowly. Suramin may be affecting newly synthesized enzyme molecules in the cytosol before they are imported into the glycosome. Suramin has also been found to affect thymidine kinase and dihydrofolate reductase. It is likely that suramin's action may be attributable to the inhibition of several of these enzymes [11]. 

 Melarsoprol is an arsenical resulting from the efforts of Ernst Freidheim in the late 1940s. His initial compound, melarsen oxide, *p*-(4,6-diamino-*s*-triazinyl-2-*yl*) aminophenylarsenoxide was complexed with dimercaptopropanol (British Anti-Lewisite) to form a less-toxic complex, merlarsoprol. Until 1990, this was the only agent available for treatment of late-stage CNS disease both of East African and West African origin. It is usually given as two to four series of three daily I.V. injections, or a single daily injection for 10 days [8]. It is insoluble in water and must be dissolved in propylene glycol, given intravenously. For this reason, it is painful to administer and destroys veins after several applications. Toxicity is an important concern with melarsoprol. This takes the form of reactive arsenical-induced encephalopathy in 10% of treated patients, which is often followed by pulmonary edema and death in more than half these cases within 48 hour. [8, 17]. Although the mechanism of melarsoprol action has been extensively studied, it still remains unclear. Parasites exposed to low (1–10 *μ*M) levels rapidly lyse. Because the bloodstream forms are intensely glycolytic, any interruption of glycolysis or interference with redox metabolism should produce this effect. Thus a series of reports has detailed melarsoprol inhibition of trypanosome pyruvate kinase (Ki, 100 *μ*M), phosphofructokinase (Ki, <1 *μ*M), and fructose-2,6-bisphosphatase (Ki, 2 *μ*M). It is likely that the rapid inhibition of fructose 2,6,bis-phosphate production is a key factor in halting glycolysis through downregulation of pyruvate kinase [11]. Other studies indicated that melarsoprol and melarsen oxide formed adducts with trypanothione (N1,N8-bisglutathionyl spermidine), a metabolite unique to trypanosomes and believed to be responsible for the redox balance of the cell and detoxification of peroxides [12, 18]. The melarsen–trypanothionine adduct (Mel T) inhibits trypanothione reductase, which has been attributed to the mode of action [19, 20]. However, melarsoprol and related arsenicals may also bind to other sulfhydryl-containing agents in the cell, including dihydrolipoate and the closely adjacent cysteine residues of many proteins. Similar to pentamidine and diminazene, melarsoprol uptake into African trypanosomes has been attributed to the P2 purine nucleoside transporter; thus, significant levels can be concentrated in the cell from a low external (plasma) concentration [12, 13]. Although most laboratory-generated melarsoprol-resistant strains have lost or modified the P2 transporter, clinical isolates appear to have retained uptake capacity [14]. 

 DFMO is the most recently developed agent for late-stage *T. b. gambiense * sleeping sickness. DFMO is an enzyme-activated irreversible inhibitor of ornithine decarboxylase (ODC), the initial enzyme in the polyamine synthetic pathway. This agent was initially developed as an antitumor agent by Merrell-Dow in the late 1970s and underwent extensive clinical trials before testing against trypanosomes, after initial testing in mouse model *T. b. brucei* infections [21]. DFMO was studied extensively in human trials in Africa [22, 23]. The standard treatment regimen resulting from the trials indicate that DFMO is <95% active when given intravenously 400 mg/kg I.V., given every 6 hours. for 14 days. DFMO cured children, adults, patients with melarsoprol-refractory strains, and patients with late-stage disease [17, 24] The short plasma half-life of DFMO necessitates constant dosing when given as an I.V. drip. The most frequent toxic reaction was reversible bone marrow suppression, which was alleviated upon reduction of the doses. The major drawbacks with respect to DFMO are its cost, the duration of treatment, and its availability [25]. DFMO rapidly and irreversibly binds to the catalytic site (cysteine 360) in mouse ODC, inactivating it. In culture, it blocks division of bloodstream trypanosomes, but it is not trypanocidal. In laboratory infections, DFMO cures when administered continuously in the drinking water as a 2% solution. Within 48 hours of administration, DFMO reduces putrescine levels to zero, and reduces spermidine levels by >75% [26]. Trypanothione levels are also significantly reduced [19]. As noted, DFMO is not trypanocidal and depends on a functional immune system to rid the host of non-dividing forms [27]. Morphologically, trypanosomes exposed to DFMO have multiple kinetoplasts and nuclei as well as forms resembling “stumpy” blood forms [26]. DFMO is curative for laboratory infections of *T. b. brucei * and *T. b. gambiense, * but not to all strains of *T. b. rhodesiense* [28]*. *The reason for this selectivity is not completely evident, although it is not due to uptake of DFMO, because it enters by passive diffusion, not transport [27, 29]. Iten et al 1997 [30] have found that *T. b. rhodesiense* isolates have an ODC with a shorter half-life than *T. b. gambiense*, which could result in lowered susceptibility to DFMO. Also levels of AdoMet are highly elevated in DFMO-treated susceptible *T. b. rhodesiense* strains, but less so in refractory isolates [25]. This elevation is the result of the block in putrescine synthesis and the resulting inability to make spermidine. The elevated level of AdoMet is the result of an AdoMet synthase insensitive to its product. DFMO treatment leads to intracellular concentrations of >5 mM, an increase of >50-fold over untreated parasites [25]. Trypanosome ODC is missing the C-terminal PEST sequence in both procyclic and bloodstream trypanosomes, and this appears to be the major reason for the stability of the trypanosome enzyme. The remainder of the ODC molecule has ~60% sequence identity with the mammalian enzyme, including a cysteine 360 residue at the demonstrated DFMO-binding site for the mammalian enzyme [31, 32]. Beyond this, trypanosomes lack a polyamine oxidase, which in mammalian cells converts spermine to the biologically active spermidine. Trypanosomes are also limited in their ability to transport putrescine and spermidine [25].

## 3. DFMO Drug Combinations

Eflornithine is the only new agent developed in 58 years for clinical use of second stage HAT. With relatively minor and irreversible side effects as compared to melarsoprol, it is superior in efficacy to melarsoprol [33, 34]. In laboratory model infections of *T. b. brucei*, DFMO was curative in combination with many new agents as well as clinically-used trypanocides, including suramin and melarsoprol [35, 36]. In the laboratory, these combinations resulted in significant reduction in DFMO dosage and time of administration [37]. Recent clinical studies have investigated the use of DFMO in combination with other clinically used trypanocides [17, 34]. Because of the recent availability of DFMO as a result of advocacy campaigns by Medicines Sans Frontiers and other organizations, coupled with financial support by Sanofi-Aventis, DFMO has been increasingly available in the field since 2001 [8]. Initial clinical combination studies [17] showed that DFMO + nifurtimox was far superior to DFMO + melarsoprol and melarsoprol + nifurtimox. The DFMO + nifurtimox regimen (NECT regimen) allowed reduction in DFMO regimen from 14 to 7 days (56 versus 28 infusions) with a 94% cure rate. Nifurtimox was given orally for 10 days. The study has been confirmed in another clinical study with a DFMO-Nifurtimox cure rate of 100% [24]. In another related study, total doses of DFMO were reduced to 14 (two/day for 7 days) and a 94% cure rate resulted with a DFMO-nifurtimox regimen [34]. All of the DFMO-nifurtimox regimens were associated with significantly reduced adverse side effects as compared to melarsoprol-based therapy [8]. The biochemical basis for this therapy most likely lies in the ability of DFMO to reduce trypanothione levels and resistance to oxidative stress [19, 20] and the ability of nifurtimox to generate oxidative stress in trypanosomes [38]. In addition to MSF, Drugs for Neglected Diseases initiative (DNDi), the Swiss Tropical Institute, Epicenter, and The World Health Organization, have collaborated in making these combination clinical trials possible (HAT-NECT Phase III Study: [9]).

## 4. Prophylaxis and Prevention

Trypanosomiasis causes complex public health and epizootic problems in many developing countries in Africa. Control programs concentrating on the eradication of vectors and drug treatment of infected people and animals have been in operation in some areas for decades. Considerable progress has been made in a number of regions, but the lack of agreement on the best approach to solving the problem of African trypanosomiasis, combined with a paucity of resources, stands in the way of effective control. Individuals can reduce their risk of becoming infected with trypanosomes by avoiding tsetse fly-infested areas, by wearing clothing that reduces the biting of the flies, and by using insect repellants. Chemoprophylaxis with suramin or pentamidine can be effective, but it is not clear which populations should use this as a preventive measure. No vaccine is available to prevent the transmission of the parasites.
